# AtAUGs Suppress the Expression of PP2C Genes to Redundantly Regulate ABA Responses in Arabidopsis

**DOI:** 10.3390/plants15071028

**Published:** 2026-03-26

**Authors:** Xutong Wang, Kaijie Zheng, Ruqian Su, Wei Wang, Xiaoxiao Jing, Yating Wang, Yaowen Wu, Nini Cheng, Siyu Chen, Shucai Wang

**Affiliations:** 1 Laboratory of Plant Molecular Genetics & Crop Gene Editing, School of Life Sciences, Linyi University, Linyi 276000, China; wangxutong0019@163.com (X.W.); 13290200167@163.com (R.S.); wangwei220201@163.com (W.W.); jingxiaoxiao86@163.com (X.J.); wuyaowen0728@163.com (Y.W.); chengnini@lyu.edu.cn (N.C.); 2 The Plant Chemetics Laboratory, Department of Biology, University of Oxford, Oxford OX1 3RD, UK; zhengkaijie@iga.ac.cn; 3 Department of Biochemistry, Microbiology and Immunology, University of Saskatchewan, Saskatoon, SK S7N 5E5, Canada; 4 Heilongjiang Provincial Key Laboratory of Environmental Microbiology and Recycling of Argo-Waste in Cold Region, College of Life Science and Biotechnology, Heilongjiang Bayi Agricultural University, Daqing 163319, China; wangyt814@nenu.edu.cn

**Keywords:** AtAUGs, abscisic acid, transcription factor, gene editing, Arabidopsis

## Abstract

The modulation of plant responses to abscisic acid (ABA) and/or abiotic stresses can be manipulated by the expression of ABA-responsive genes, which is affected by phytohormone ABA. While some ABA-responsive genes have been shown to regulate plant responses to ABA and/or abiotic stresses, the functions of numerous ABA-responsive genes remain unknown. Therefore, characterizing these unstudied genes would provide a practical way to identify novel regulators of plant adaptations to ABA and/or abiotic stresses. Here, we characterized four closely related unstudied ABA-responsive genes in *Arabidopsis thaliana*, named *Arabidopsis thaliana ABA-up regulated genes* (*AtAUGs*). We found that ABA treatment induces *AtAUGs* expression level, and our results in transfected protoplasts show that AtAUGs exhibit nucleus localization and downregulate the co-transfected reporter expression level. The results of ABA sensitivity assays, including seed germination, cotyledon greening, and root extension assay show that transgenic plants overexpressing *AtAUGs* had increased sensitivity, but *ataugs* mutants generated by isolating T-DNA insertion lines or through CRISPR/Cas9 gene-editing of *AtAUGs* had decreased sensitivity; in addition, the greatest decrease in ABA sensitivity was observed in the *ataug1 ataug2 ataug3 ataug4* (*ataug1234*) quadruple mutants. The qRT-PCR results show that the expression levels of several Type 2C Protein Phosphatase (PP2C) genes, the key negative regulator genes of ABA signaling including *PP2CA*, *Hypersensitive to ABA 1* (*HAB1*), *HAB2*, *Highly ABA-Induced PP2C protein 3* (*HAI3*), *ABA-Hypersensitive Germination 1* (*AHG1*), and *ABA Insensitive 2* (*ABI2*) decreased in *35S:AtAUGs* transgenic plants, but increased in the *ataug1234* quadruple mutants. Taken together, these results suggest that *AtAUGs* are ABA-responsive genes, and AtAUGs positively regulate ABA responses in a redundant manner, by downregulating the expression of crucial negative regulator genes in ABA signaling.

## 1. Introduction

Abscisic acid (ABA) is a crucial phytohormone that functions as a specialized metabolic regulator in plants. Like other phytohormones, ABA regulates cellular metabolism under both normal and stressful conditions, and it performs multiple roles in plant growth and development, ranging from seed germination and seedling development to plant senescence [1,2,3,4]. In addition, ABA serves as a mediator of plant resilience to environmental stressors such as dehydration, cold, and salt stress [5,6,7,8].

ABA modulates plant abiotic stress responses through signal transduction to activate/repress down-stream ABA-responsive genes, and ABA signaling is mediated by the interplay of several key regulators [9,10,11,12]. Based on their functions in signaling transduction, the crucial mediators of ABA signaling can be classified as positive or negative. The Pyrabactin Resistance/PYR1 Like/Regulatory Components of ABA Receptors (PYR/PYL/RCAR) receptors, the Snf1 (Sucrose-Non-Fermentation 1)-Related Kinases Subfamily 2 (SnRK2s) protein kinases, and the down-stream ABA-Responsive Element Binding Protein/ABRE-Binding Factor/ABA Insensitive 5 (ABF/AREB/ABI5)-type bZIP transcription factors serve as positive regulators [13,14,15,16], while the Type 2C Protein Phosphatases (PP2Cs) proteins are suppressors [17,18]. Current evidence suggests that the ABA contents in plant cells are very low under normal growth conditions; therefore, PP2C proteins such as PP2CA, Hypersensitive to ABA 1 (HAB1), and HAB2 are able to bind to SnRK2 proteins, including SnRK2.2, SnRK2.3, and SnRK2.6, thereby inhibiting their activity, and blocking ABA signal transduction. Whereas, under abiotic stress conditions, ABA accumulates in plant cells, and binds to PYR/PYL/RCAR receptors such PYL4, PYL5 and PYL6, which then binds to PP2Cs; this leads to the release of SnRK2s, therefore enabling their self-activation and the phosphorylation of ABF/AREB/ABI5-type bZIP transcription factors, including ABF2, ABF3, and ABF4. Phosphorylated ABF/AREB/ABI5-type bZIP transcription factors regulate down-stream ABA-mediated genes expression, thus mediating the mechanism of plant tolerance to environmental adversity [19,20,21,22].

In line with this, altering ABA signaling regulator expression modulates abiotic stress responses in plants. For example, transgenic plants overexpressing ABA receptor genes displayed improved drought resistance [23,24], and the *SnRK* gene mutant *snrk2.2 snrk2.3 snrk2.6* and the ABF/AREB/ABI5-type bZIP transcription factor gene mutant *areb1 areb2 abf3 abf1* exhibited enhanced sensitivity to drought stress [9,19]. Some down-stream ABA-responsive genes, *AtbZIP62*, a bZIP transcription factor gene; *ABI3*, a B3 transcription factor gene; and *ABI4*, an APETALA2 (AP2) transcription factor gene have also been shown to regulate plant tolerance to environmental adversity [25,26,27,28].

While ABA-responsive genes are likely to be involved in plants response to abiotic stresses, the function of many of these genes has not yet been elucidated. Thus, it would be beneficial to identify novel ABA-responsive genes that may regulate plant environmental stress tolerance. Indeed, we have identified a few novel ABA-responsive genes which mediate plant responses to ABA and/or abiotic stresses, such as ABA Induced Transcription Repressors (AITRs), ABA-induced Serine-rich Repressors (ASRs), and ABA-inducible Signal peptide-containing DUF538 proteins (ASDs) [12,29,30,31].

In our previous transcriptome analyses of ABA-responsive genes in *Arabidopsis thaliana*, we noticed a small group of ABA-induced genes whose functions remain largely unknown. These genes attracted our attention because unidentified ABA-responsive genes may represent novel regulators in ABA signaling. Here, we reported the identification of *Arabidopsis thaliana ABA-up regulated genes* (*AtAUGs*) from ABA-responsive genes with previously uncharacterized functions. Four *AtAUGs* were identified from a transcriptome dataset as ABA induced genes, and the quantitative RT-PCR (qRT-PCR) results confirmed that they are all ABA-responsive. The protoplast transfection assays, ABA sensitivity assays and qRT-PCR analysis results indicate that AtAUGs were involved in regulating plant responses to ABA, and they act redundantly, possibly by suppressing the expression of certain negative regulators of ABA signaling.

## 2. Results

### 2.1. ABA Induces the Expression of AtAUGs

To identify novel regulators of ABA and/or abiotic stress responses, ABA-responsive genes with unknown function from an Arabidopsis transcriptome database were characterized [12]. We found that *At4g13530*/*AtAUG1*, *At4g10080*/*AtAUG2*, *At3g03870*/*AtAUG3*, and *At5g18130*/*AtAUG4*, four closely related genes with unknown function, are ABA responsive, with Fragments Per Kilobase Million (FPKM) values in ABA treated and control samples (ABA/Control) of 38.42/14.09, 42.23/24.68, 14.13/6.66, and 41.30/7.33, respectively.

**Figure 1:**
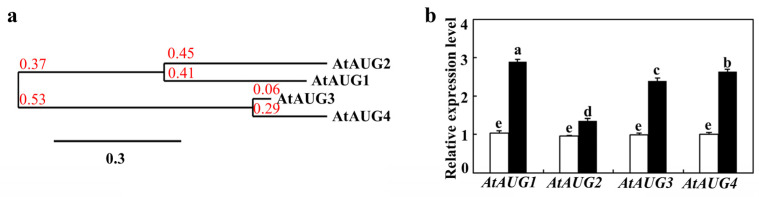
AtAUGs shares close relationship in phylogenetic tree and gene expression level is altered under ABA treatment. (**a**) Phylogenetic tree of four AtAUGs. The full amino acid sequences of four AtAUGs was used for bioinformatics analysis on the https://ngphylogeny.fr/ (accessed on 30 May 2025) with “one click” mode in default setting. Branch length is indicated as the bar. (**b**) Expression of four *AtAUGs* under ABA treatment. RNA was extracted from Col 14 days seedlings after exposure to 50 µM ABA for 4 h and used for qRT-PCR. Internal reference gene was *ACT2*. Four *AtAUGs* expression were shown by fold change relative to expression of mock-treated seedlings. Data represents the standard deviations (SDs) of three biological replicates and each experiment including three technical replicates. Different letters (a–e) indicate significant difference between different columns (*p* < 0.05), as determined by one-way ANOVA.

A phylogenetic tree constructed from alignment of full-length AtAUG amino acid sequences demonstrated that AtAUG1 and AtAUG2 form one clade, while AtAUG3 and AtAUG4 form another (Figure 1a). qRT-PCR was applied to evaluate *AtAUGs* expressions in Col seedlings treated with ABA, with the aim of verifying the ABA responses of these genes. To this end, wild-type seedlings were exposed to ABA for 4 h, and mock treated seedlings were used as a control. Following cDNA synthesis from isolated total RNA, qRT-PCR analysis with *ACTIN2* (*ACT2*) as the reference gene confirmed that *AtAUG*s expressions was upregulated by ABA, showing ~1.5–~3 fold increases in induction (Figure 1b). This validates our initial transcriptomic findings. These genes were collectively named *Arabidopsis thaliana ABA-up regulated Gene 1* (*AtAUG1*) to *AtAUG4* due to the up-regulated gene expression caused by ABA treatment.

### 2.2. AtAUGs Exhibit Transcriptional Repression Activity

**Figure 2:**
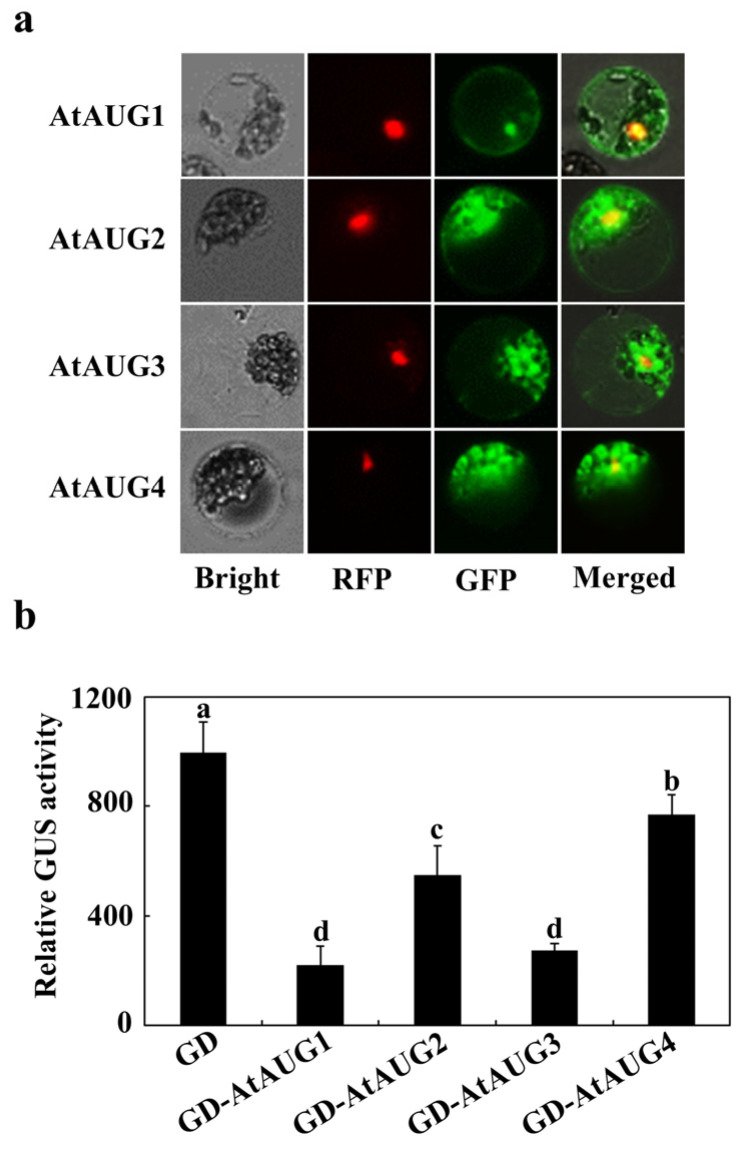
AtAUGs protein localization and transcriptional activities. (**a**) AtAUGs protein subcellular localizations. *AtAUGs-GFP* plasmids co-transfected with *NLS-RFP* plasmid into Arabidopsis protoplasts. Then transfected Arabidopsis protoplasts were incubated overnight under dark condition and fluorescence confocal microscope was used to detect the GFP and RFP signal. (**b**) AtAUGs transcriptional activities. Co-transfecting *GD-AtAUGs* plasmids or control plasmid *GD* with the activator plasmid *LD-VP* and the reporter plasmid *LexA-Gal4:GUS* into Arabidopsis protoplasts. After incubating overnight under dark condition, the GUS activities of transfected Arabidopsis protoplasts were measured. Data represents SDs of three biological replicates and each experiment including three technical replicates. Different letters (a–d) indicate significant difference between different columns (*p* < 0.05), as determined by one-way ANOVA.

According to the Subcellular Localisation Database for Arabidopsis Proteins (https://suba.live/, accessed on 3 June 2025), all of the studied AtAUGs are located in nucleus. To verify this, we observed the subcellular localization of AtAUGs in transfected protoplasts by isolating AtAUGs-GFP plasmids and transfecting them into Arabidopsis protoplasts derived from rosette leaves. Plasmid DNA of the nuclear indicator NLS-RFP construct was co-transfected [32], and the GFP and RFP fluorescence of transfected protoplasts were observed after incubation for 20–22 h. As shown in Figure 2a, AtAUG1-GFP fluorescence primarily localizes in nucleus, and the fluorescence of the other AtAUGs-GFP was examined in the nucleus and other parts of the cells.

Based on the finding that AtAUGs localize to the nucleus, though not exclusively, we next assessed their transcriptional activity using transient transfection assays in Arabidopsis protoplasts. To this end, we performed a transient expression assay in Arabidopsis leaf protoplasts. The *GD-AtAUGs* fusion constructs, *LD-VP* activator and *LexA-Gal4:GUS* reporter were co-transfected. As a control, the GD plasmid alone was co-transfected with the same activator and reporter. Figure 2b shows that while LD-VP strongly activated reporter gene expression, co-transfection of *GD-AtAUGs* significantly repressed this activation. These results suggest that AtAUGs exhibit transcriptional repression activity.

### 2.3. AtAUGs Positively Regulate Plant Responses to ABA

**Figure 3:**
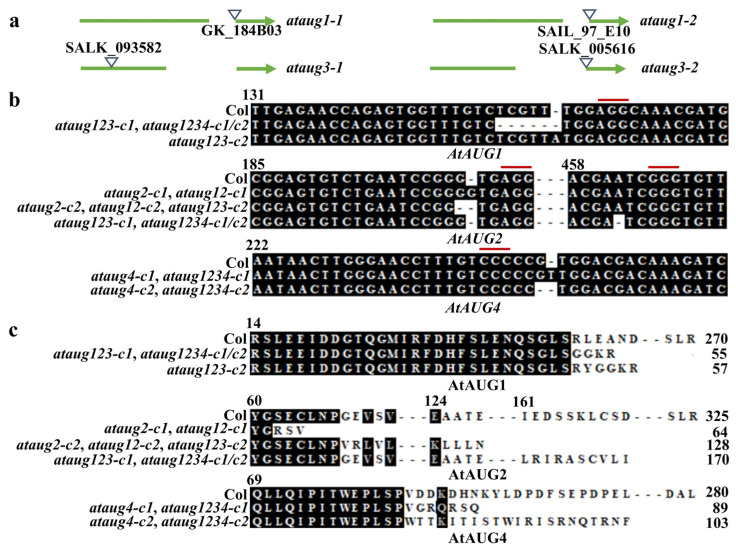
Generation of *ataugs* mutants. (**a**) T-DNA insertion sites in the *ataug1-1*, *ataug1-2*, *ataug3-1* and *ataug3-2* single mutants. Seeds of the T-DNA insertion lines were obtained from ABRC, and homozygous mutants were screened via PCR analysis. (**b**) Nucleotide sequence alignment of the *AtAUGs* in the Col and the *ataug2* and *ataug4* single, *ataug12* double, *ataug123* triple, and *ataug1234* quadruple mutants. Red lines indicate proto-spacer adjacent motifs (PAMs). Numbers show positions of the nucleotide from the start codon. (**c**) Amino acid alignments of AtAUGs in the Col and the *ataug2* and *ataug4* single, *ataug1 ataug2* (*ataug12*) double, *ataug1 ataug2 ataug3* (*ataug123*) triple, and *ataug1 ataug2 ataug3 ataug4* (*ataug1234*) quadruple mutants. Open-reading frames (ORFs) of *AtAUGs* in the mutants were analyzed by https://www.ncbi.nlm.nih.gov/orffinder/, accessed on 12 October 2024. Predicted AtAUGs amino acid sequences were aligned with the sequences in Col. Numbers above the sequences show position of the amino acid from the first M amino acid, and numbers on the right of the sequences show the total numbers of amino acids.

Considering that *AtAUGs* are ABA responsive genes (Figure 1b), both loss-of-function mutants and overexpression transgenic plants were utilized to examine the role of AtAUGs in ABA response. Homozygous *ataug1-1* and *ataug1-2* mutants were isolated from T-DNA insertion lines available for *AtAUG1* (Figure 3a). To generate *AtAUG1* overexpression transgenics, Col plants were transformed with *35S:AtAUG1* construct in the *pPZP211* vector. Single T-DNA insertion transgenic lines were first selected from T2 generation, and then plants with homozygous overexpression were derived from the T3 generations.

**Figure 4:**
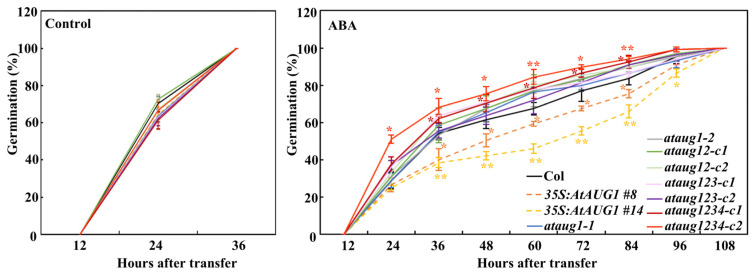
Seeds germination rates of the Col, the *35S:AtAUG1* transgenic plants, *ataug1* single, *ataug12* double, *ataug123* triple and *ataug1234* quadruple mutants under control or ABA treatment. 40 seeds were plated on 1/2 MS medium ± 1 µM ABA after surface-sterilization, and kept for 2 days in the dark cool room at 4 °C. Then seeds were transferred to 22 °C room, and germination was recorded every 12 h. Data represents SDs of three biological replicates. Each replicate containing 40 seeds for each line and each experiment including three technical replicates. * Significantly different from the Col (Student’s *t*-test, * *p* < 0.05, ** *p* < 0.01).

The ABA response phenotypes of the *ataug1* mutants and *35S:AtAUG1* transgenic plants were characterized using three standard assays: seed germination assay, cotyledon greening assay, and root extension assay. For seed germination assays, all seeds on control plates germinated within 36 h in the growth chamber. In contrast, on the plates with ABA, *35S:AtAUG1* transgenic plants exhibited a reduction in the rate of seedling germination, which was significantly lower than that of Col. In addition, *ataug1* mutants displayed an indistinguishable germination rate from Col (Figure 4).

**Figure 5:**
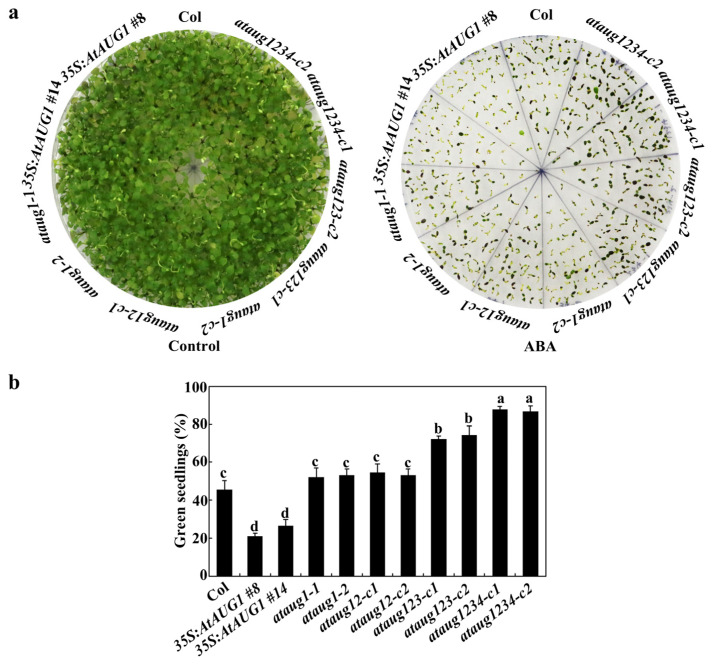
Effects of ABA on cotyledon greenings of the Col, the *35S:AtAUG1* transgenic plants, *ataug1* single, *ataug12* double, *ataug123* triple and *ataug1234* quadruple mutants. (**a**) Image of seedlings on plates under ABA treatment. 40 seeds were plated on 1/2 MS medium ± 1 µM ABA after surface-sterilization, and kept for 2 days in the dark cool room at 4 °C. Then seeds were transferred to 22 °C room, and images were taken after 18 days. (**b**) Green seedlings percentage under ABA treatment. The number of green seedlings was counted 18 days after transferred and results was used to calculate the percentage of green seedlings. Data represents SDs of three biological replicates. Each replicate containing 40 seeds for each line and each experiment including three technical replicates. Different letters (a–d) indicate significant difference between different columns (*p* < 0.05), as determined by one-way ANOVA.

**Figure 6:**
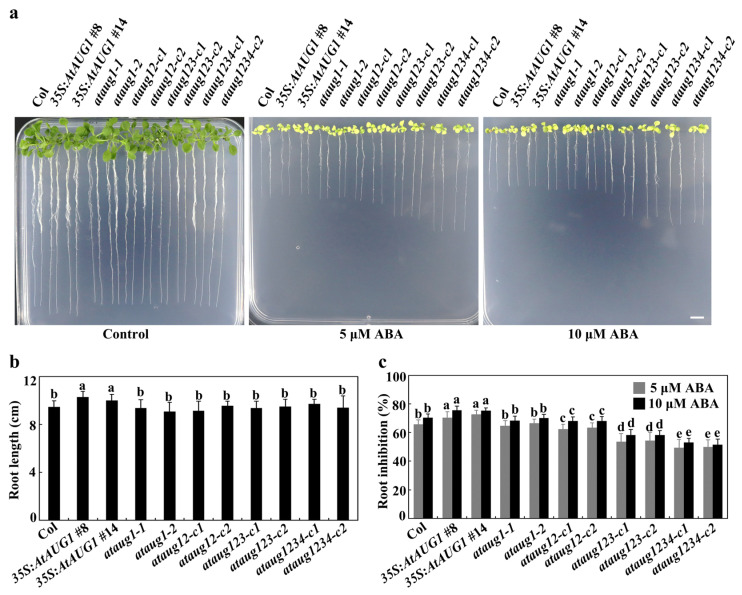
Root elongation of the Col, the *35S:AtAUG1* transgenic plants, *ataug1* single, *ataug12* double, *ataug123* triple and *ataug1234* quadruple mutants under ABA treatment. (**a**) Image of seedlings on plates under ABA treatment. At least 100 seeds were plated on 1/2 MS medium after surface-sterilization, and kept for 2 days in the dark cool room at 4 °C. Then seeds were transferred to 22 °C room, and grew vertically for 3 days. After that, at least 18 seedlings were transferred to plates contain 0 μM, 5 μM and 10 μM ABA. Images were taken 10 days after transferred. Scale bar = 1 cm. (**b**) Primary root length of the plants. Root length of 13-day-old seedlings was measured and calculated. Data present mean ± SD of 18–21 seedlings. (**c**) Percentage of root inhibition by 5 μM and 10 μM ABA. Length of 10 days newly elongated root after the transfer was measured, and the percentage of ABA-mediated inhibition was calculated. Data represents SDs of three biological replicates. Each replicate containing at least 18 seeds for each line and each experiment including three technical replicates. Different letters (a–e) indicate significant difference between different columns (*p* < 0.05), as determined by one-way ANOVA.

In cotyledon greening assays, no differences were observed in the *ataug1* mutants, *35S:AtAUG1* transgenic plants, and Col plants on control plates, but on the plates with ABA, the *35S:AtAUG1* transgenic plants produced much fewer green seedlings, and the *ataug1* mutants produced slightly increased numbers of green seedlings (Figure 5a). The quantitative results show that the *35S:AtAUG1* transgenic plants produced 22% green seedlings, and the *ataug1* mutants produced 52%, while Col plants produced 46% (Figure 5b). In the root extension assays, we revealed that on the control plates, the *35S:AtAUG1* transgenic seedlings produced relatively longer roots with 10.2 cm than the Col seedlings with 9.3 cm (Figure 6a), while the root length of the *ataug1* mutant seedlings was similar to that of the Col seedlings (Figure 6b). On the plates with 5 μM ABA or 10 μM ABA, the *35S:AtAUG1* seedlings produced shorter roots than those on the control plates (Figure 6a), while the root length of *ataug1* mutants was not significantly different from that of Col seedlings on the ABA plates (Figure 6a). The quantitative results showed that a 6% increase in root inhibition percentage in the *35S:AtAUG1* transgenic plant seedlings of ABA-mediated root inhibition compared to wild-type seedlings (Figure 6c).

**Figure 7:**
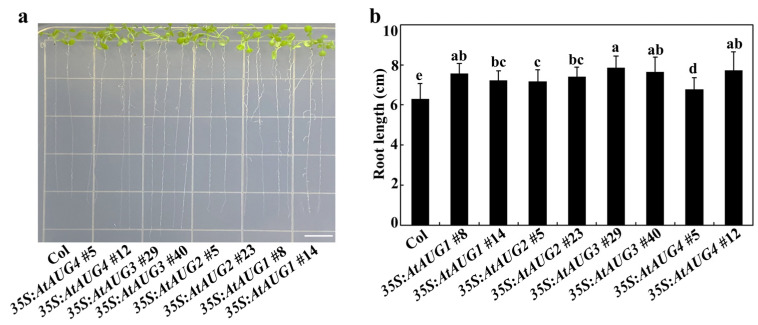
Primary roots of the Col and *35S:AtAUGs* transgenic plants. (**a**) Image of seedlings on plates. At least 100 seeds of the Col, and the *35S:AtAUGs* transgenic plants were sown on plates with 1/2 MS medium after surface-sterilization. Plates were kept for 2 days in the dark cool room at 4 °C. Then plates were transferred to 22 °C room and grow vertically. After 8 days transfer the images of seedings were captured. Scale bar = 1 cm. (**b**) Primary root length of the plants. Measured and recorded the 8-day-old seedlings root length. Data represents SDs of three biological replicates. Each replicate containing at least 18 seeds for each line and each experiment including three technical replicates. Different letters (a–e) indicate significant difference between different columns (*p* < 0.05), as determined by one-way ANOVA.

These results indicate that AtAUG1 positively regulates plant responses to ABA. *AtAUG2*, *AtAUG3* and *AtAUG4* overexpression transgenic plants were created by transforming the corresponding *35S:AtAUGs* construct in *pPZP211* into wild-type plants, with the aim of verifying whether other *AtAUGs* have similar functions. The *35S:AtAUG2*, *35S:AtAUG3*, and *35S:AtAUG4* transgenic seedlings exhibited longer roots compared to the Col wild-type seedlings, a phenotype similar to that observed in the *35S:AtAUG1* seedlings (Figure 7a), with lengths of 6.8–7.9 cm for the transgenic plant compared to 6.3 cm for the Col wild-type plant (Figure 7b). We also generated single mutants for *AtAUG2*, *AtAUG3*, and *AtAUG4*. As T-DNA insertion lines were available for *AtAUG3*, we isolated the homozygous *ataug3-1* and *ataug3-2* mutants from the T-DNA insertion lines (Figure 3a). Whereas no T-DNA insertion lines were available for *AtAUG2* and *AtAUG4*, we generated *ataug2-c1*, *ataug2-c2*, *ataug4-c1*, and *ataug4-c2* mutants through gene editing by transforming the Col plants possessing *pHEE401E* constructs with specific target sequences, and identifying homozygous mutants based on amplification and sequencing of the corresponding *AtAUG* genes, along with detection of the Cas9 transgene. The target sites of all *ataug2* and *ataug4* single mutants were found to be single-nucleotide insertion or deletion sites (Figure 3b). Consequently, these kinds of frameshift mutations led to amino acid substitutions and premature termination of AtAUGs proteins (Figure 3c).

The obtained *35S:AtAUG2*, *35S:AtAUG3*, and *35S:AtAUG4* transgenic plants and *ataug2*, *ataug3*, and *ataug4* single mutants were then subjected to ABA sensitivity assays, including seed germination and cotyledon greening tests. In the seed germination assays, all *AtAUG*-overexpressing lines exhibited significantly reduced germination rates following ABA treatment, similarly to the *35S:AtAUG1* lines (Figure S1a). In contrast, the germination rates of the *ataug* single mutants were indistinguishable from those of wild-type plants (Figure S2a). In cotyledon greening assays, all the *35S:AtAUGs* transgenic plants produced fewer green seedlings (Figure S1b), with a 10–38% decrease (Figure S1c), while all the single mutants produced more green seedlings (Figure S2b), with an increase of 2–15% (Figure S2c).

### 2.4. Redundant Functions of AtAUGs in Modulating Plant Responses to ABA

Having shown that all of the studied AtAUGs enhance plant responses to ABA and demonstrate transcriptional repression activity, we aimed to explore whether these genes perform redundant functions. To this end, we generated *ataug12* double, *ataug123* triple, and *ataug1234* quadruple mutants. The *ataug12* double mutants were generated by transforming *ataug1-1* with the *pHEE401E-AtAUG2* construct; the *ataug123* triple mutants were generated by transforming *ataug3-2* with the *pHEE401E-AtAUG1 AtAUG2* construct; and the *ataug1234* quadruple mutant was generated by transforming isolated Cas9-free *ataug123-c1* with the *pHEE401E-AtAUG4* construct. All obtained mutant lines, whether single nucleotide alternations or microdeletions, occurred at the modification sites of the corresponding genes (Figure 3b), resulting in substitution of amino acids and premature stops in the AtAUGs proteins (Figure 3c).

The obtained *ataug12* double, *ataug123* triple and *ataug1234* quadruple mutants were then subjected to ABA sensitivity assays. In the seed germination assays, all mutants showed increased germination rates, with the *ataug1234* quadruple mutants exhibiting the highest rate (Figure 4). In the cotyledon greening assays, the *ataug123* triple and *ataug1234* quadruple mutants produced much more green seedlings (Figure 5a), and the quantitative results show that the *ataug123* triple and *ataug1234* quadruple mutants produced 73% and 87% green seedlings, compared to 53% in the *ataug12* double and 52% in the *ataug1* mutants, and 45% in the wild-type plants (Figure 5b). In the root elongation assays, we found that on the control plates, all the mutant seedlings produced a similar root length to that of the Col seedlings (Figure 6a,b), while on the plates with either 5 μM or 10 μM ABA, the *ataug123* triple and *ataug1234* quadruple mutants produced much longer roots (Figure 6a). The quantitative results show that root inhibition percentage decreased gradually from the *ataug12* double mutants to the *ataug1234* quadruple mutants, with 53% inhibition in the *ataug1234* quadruple mutants compared to 70% in the Col wild type seedlings (Figure 6c).

### 2.5. AtAUGs Affects the Expression of Some PP2C Genes in ABA Signaling

**Figure 8:**
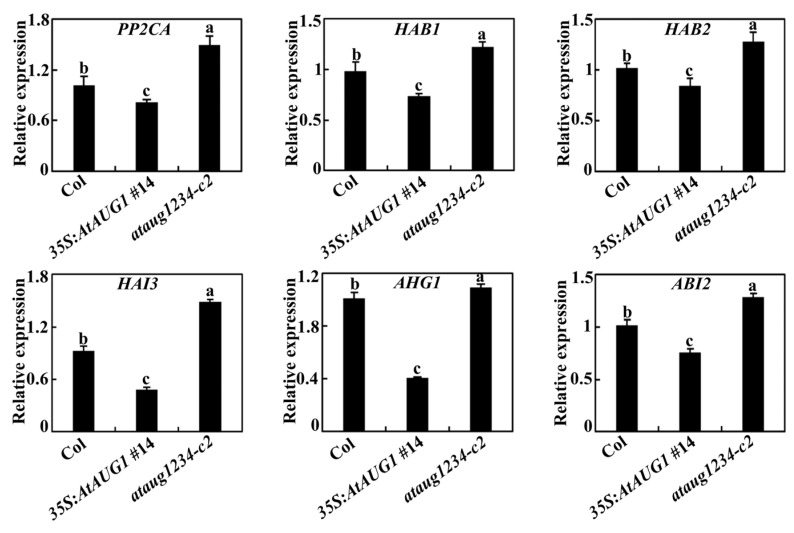
Relative expression levels of the PP2C genes, including *PP2CA*, *HAB1*, *HAB2*, *HAI3*, *AHG1* and *ABI2* in the Col, the *35S:AtAUG1* transgenic plants, and the *ataug1234* quadruple mutants. RNA was extracted from the plants and qRT-PCR was conducted to test the expression levels of PP2C genes. Internal reference gene was *ACT2*. Data represents SDs of three biological replicates and each experiment including three technical replicates. Different letters (a–c) indicate significant difference between different columns (*p* < 0.05), as determined by one-way ANOVA.

The results described above demonstrate that *AtAUGs* redundantly and positively regulate ABA responses in Arabidopsis. To further elucidate the mechanism that how *AtAUGs* modulate plant responses to ABA, we investigated whether they affect the transcript level of crucial genes involved in the ABA signaling pathway. Against this background, the expression of key genes regulating ABA signaling in the Col plants, *35S:AtAUG1* transgenic plants, and *ataug1234* quadruple mutants was analyzed through qRT-PCR, with *ACT2* employed as an internal reference. Based on finding that AtAUG1 localizes primarily in the nucleus and exhibits transcriptional repression activity (Figure 2), ABA sensitivity was elevated in the *35S:AtAUG1* transgenic plants, whereas it was reduced in the *ataug1234* quadruple mutants (Figure 4, Figure 5 and Figure 6). We focused on characterizing genes whose expression was down-regulated in the *35S:AtAUG1* transgenic plants but up-regulated in the *ataug1234* quadruple mutants, and found that the expression of several PP2C genes including *PP2CA*, *HAB1*, *HAB2*, *HAI3*, *AHG1* and *ABI2*, was upregulated in the *ataug1234* quadruple mutants, but downregulated in the *35S:AtAUG1* transgenic plants (Figure 8). We also found that the expression of these PP2Cs genes was down-regulated in *35S:AtAUG2*, *35S:AtAUG3*, and *35S:AtAUG4* transgenic plants (Figure S3).

## 3. Discussion

ABA mediates plants’ adaptive responses to environmental stresses, and also influence plant growth and development via a signaling transduction pathway that activates or suppresses ABA-responsive gene expression [4,5,6,7]. Consistent with this, mutation and/or overexpression of key ABA signaling regulators, including PYR/PYL/RCAR receptors, SnRK2 kinases, and ABF/AREB/ABI5-type bZIP transcription factors, as well as some down-stream ABA-responsive genes such as *AtbZIP62*, *ABI3* and *ABI4* affected plant responses to abiotic stresses [23,25,27,28]. Through the functional characterization of ABA-responsive genes with previously unknown functions, we already have identified a few novel regulator genes with ABA responses, including *AITRs*, *ASRs*, and *ASDs* [29,30,31]. In particular, AITRs have been characterized as negative regulators of abiotic stress responses in plants. Consequently, they have been targeted using CRISPR/Cas9 editing to enhance abiotic stress tolerance in crops such as soybean and tobacco [33,34,35].

In this study, we demonstrated that AtAUGs identified from ABA-responsive genes with unknown function are involved in regulating plant responses to ABA. First, we discovered that these genes are ABA-responsive from a transcriptome data set, a finding which was subsequently verified by qRT-PCR assays showing their upregulation upon ABA treatment (Figure 1). Second, in the seed germination assays, cotyledon greening assays and root inhibition assays, we found that ABA sensitivity was increased in the transgenic plants overexpressing *AtAUGs*, but decreased in *ataugs* mutants, with the strongest decrease observed in the *ataug1234* quadruple mutants (Figure 4, Figure 5 and Figure 6, Figures S1 and S2). In addition, we found that the transgenic plants overexpressing *AtAUGs* produced longer roots (Figure 6 and Figure 7). These results indicate that AtAUGs positively and redundantly regulate ABA responses in Arabidopsis. Third, although our transient expression assays in protoplasts did not show predominant nuclear localization of AtAUGs (Figure 2a), somehow which differs from the data in the Subcellular Localisation Database for Arabidopsis Proteins (https://suba.live/, accessed on 3 June 2025), the expression of co-transfected *LexA-Gal4:GUS* reporter gene was found to be repressed by AtAUGs (Figure 2b). Consistent with this, the expression of PP2C genes, including *PP2CA*, *HAB1*, *HAB2*, *HAI3*, *AHG1*, and *ABI2*, was reduced in the *35S:AtAUGs* transgenic plants but elevated in the *ataug1234* quadruple mutants (Figure 8 and Figure S3), suggesting that AtAUGs negatively regulate the genes of the key regulator PP2C in ABA signaling to modulate plant ABA responses. However, it is still unclear whether PP2C genes are direct targets of AtAUGs, and if they are, how AtAUGs regulate PP2C genes expression. And important directions for future studies include analysis of the gene expression of ABA downstream markers in *35S:AtAUGs* transgenic plants and *ataug1234* quadruple mutants; epistasis experiments; and studies on the genetic interaction between *ataug1234* quadruple mutants and known ABA signaling mutants. Such investigations would clarify whether AtAUGs act upstream of, act parallel to, or intersect with the PYL-PP2C-SnRK2-ABF/ABI5 module, thereby providing a more complete understanding of their regulatory roles in ABA responses.

ABA modulates plant responses to abiotic stresses through a signaling pathway, that activates or represses ABA-responsive genes [7,11,21]. Therefore, the expression of key ABA signaling regulators and downstream responsive genes is a critical factor influencing plant abiotic stress tolerance [9,19,23,24]. Our data indicates that *AtAUGs* are ABA-responsive (Figure 1), and are therefore involved in modulating ABA responses in Arabidopsis (Figure 4, Figure 5 and Figure 6, Figures S1 and S2). A crucial goal in future research is verifying whether AtAUG play a role in abiotic stress and whether AtAUG homologs in crops have similar functions in ABA responses and abiotic stress. Furthermore, future studies should target for molecular breeding to improve abiotic stress tolerance in crops.

Our transient expression assays in protoplasts showed that AtAUG1 was primarily located in the nucleus, whereas other AtAUGs did not show predominant nuclear localization (Figure 2a). This multi-compartment localization suggests that AtAUGs may not only function as nuclear regulators. Dual or multiple subcellular localization is also a feature of regulatory proteins involved in ABA signaling pathways [36]; and the presence of AtAUGs proteins outside the nucleus may imply additional regulatory roles. Further investigation will be required to determine whether AtAUGs localization is dynamically regulated under ABA treatment. Nonetheless, the predominant nuclear localization observed in this study supports AtAUGs’ potential involvement in transcriptional regulation, while the additional cytoplasmic signals highlight the possibility of broader regulatory functions.

Our findings reveal that AtAUGs act redundantly to modulate ABA signaling in Arabidopsis (Figure 4, Figure 5 and Figure 6), and although overexpression of each gene resulted in increased root length, their functional redundancy might not mediate root elongation (Figure 6 and Figure 7). As the root length produced by the single, double, triple, and quadruple mutant seedlings was indistinguishable from that of the Col seedlings (Figure 6), it is possible that *AtAUGs* overexpression repress some root elongation repressor genes; conversely, the knockout of all *AtAUGs* did not lead to an increase in their expression. We also noticed that *35S:AtAUGs* transgenic plants showed increased root growth under control conditions, which was attributed to higher ABA sensitivity under ABA treatment. One possible explanation is that *AtAUGs* overexpression may alter basal ABA signaling sensitivity rather than constitutively activating ABA responses. Under control conditions, modulation of *PP2Cs* expression could shift the signaling balance, potentially affecting growth-regulatory pathways without triggering full ABA-mediated inhibition. However, upon exogenous ABA application, the altered signaling level may lead to an enhanced response, resulting in increased ABA sensitivity. In addition, ABA signaling is known to interact extensively with other hormone pathways, including auxin and gibberellin, both of which play central roles in root growth regulation [37,38]. Therefore, the growth promotion observed under control conditions may reflect indirect effects mediated through hormonal crosstalk rather than a simple relationship with ABA signaling.

## 4. Materials and Methods

### 4.1. Plant Materials and Growth Conditions

The wild-type control in this study was the Columbia-0 (Col) ecotype of Arabidopsis (*Arabidopsis thaliana*). Seeds of GK_184B03, SAIL_97_E10, SALK_093582, and SALK_005616 were obtained from the Arabidopsis Biological Resource Center (ABRC), and were used to isolate *ataug1-1*, *ataug1-2*, *ataug3-1*, and *ataug3-2*, respectively. The *ataug12* double, *ataug123* triple, and *ataug1234* quadruple mutants were created through CRISPR/Cas9 gene editing; and all *AtAUGs* overexpression lines were created in the Col background. To obtain Arabidopsis plants used for protoplast isolation and plant transformation, Col seeds were directly sown into soil and grown in growth chambers. The seeds used for ABA treatment, ABA sensitivity assays, and RNA isolation were placed in a centrifuge tube and sterilized with bleach for 10 min. Then, the seeds were rinsed with sterilized water 4 times before adding 0.1% agar, and were sown on plates containing 1/2 Murashige and Skoog (MS) medium. Following 48 h incubation at 4 °C, these plates were transferred to a growth chamber, then incubated inside the growth chamber under long-day conditions (16 h light/8 h dark) at 22 °C.

### 4.2. RNA Extraction, RT-PCR, and qRT-PCR Analysis

Fourteen-day-old seedlings of Col, the *35S:AtAUG1* transgenic plants, the *ataug1234* quadruple mutants were collected and total RNA was extracted using an Easy Pure plant RNA kit (TransGen Biotech, Beijing, China). First-strand cDNA was synthesized using an EasyScript First-strand DNA Synthesis Super Mix kit (TransGen Biotech, Beijing, China), and the *ACT2* gene was served as an internal control gene for qRT-PCR. Previous studies have reported the primers for *ACT2* and *PP2Cs* [12], and the *AtAUGs* specific primers are listed in Table S1. The qRT-PCR reactions were performed with three biological and technical replicates, and then analyzed using the 2^−∆∆CT^ method.

### 4.3. Constructs

The constructs of *LexA-Gal4:GUS*, *LD-VP*, and *GD*, as well as the nuclear indicator *NLS-RFP*, have been described previously [32,39].

To generate *GD-AtAUGs* constructs for the transcriptional activity assay, the full-length coding sequence (CDS) of *AtAUGs* was amplified via RT-PCR using RNA extracted from Col seedlings as the template. Following digestion with suitable restriction enzymes, the product was fused with GD and subsequently cloned in frame into the *pUC19* vector, driven by the *CaMV 35S* promoter.

In the construction of the *AtAUGs-GFP* for subcellular localization assays, the full-length CDS of *AtAUGs* lacking a stop codon was amplified; it was then digested and cloned in-frame with GFP into the *pUC19* vector under the control of the *CaMV 35S* promoter.

To generate *35S:AtAUGs* constructs for plant transformation, the full-length CDS of *AtAUGs* was amplified by RT-PCR. The products were then digested with proper restriction enzymes and inserted in-frame into the *pUC19* vector with an N-terminal *HA* tag, under the control of the *CaMV 35S* promoter. Then constructs were digested with proper restriction enzymes and subcloned into the binary vector *pPZP211*. The primers used to generate the *35S:AtAUGs* constructs are listed in Table S1.

In the construction of CRISPR/Cas9 for *AtAUGs* gene editing, target sequences of *AtAUG1*, *AtAUG2*, *AtAUG3*, and *AtAUG4* were selected using CRISPRscan (https://www.crisprscan.org/, accessed on 12 October 2022) and then evaluated with Cas-OFFinder (http://www.rgenome.net/cas-offinder/, accessed on 12 October 2022). The sequences selected were as follows: for *AtAUG1*, GAGTGGTTTGTCTCGTTTGG(AGG); for *AtAUG2*, (AGG)CACGGTGAGAACGAATCGGG and GGGTCGGAGTGTCTGAATCC(GGG); and for *AtAUG4*, (CCC)CCGTGGACGACAAAGATCAC and CCGGATTTCTCGGAACCAGA(CCC). The target sequences were cloned into *pHEE401E* using the method employed in [29]. The primers used to generate the CRISPR/Cas9 constructs are listed in Table S1.

### 4.4. Generation of Transgenic Plants, and Cas9-Free Mutant Isolation

To screen *AtAUGs* overexpressing plants and Cas9-free *ataug2*, and *ataug4* single mutants, the *pPZP211-35S:AtAUGs*, *pHEE401E-AtAUG2*, *pHEE401E-AtAUG4* constructs were transfected into *Agrobacterium tumefaciens* strain GV3101 and used to transform 5-week-old Col plants using the floral dip method [40]. The *pHEE401E-AtAUG2* construct was introduced into the *ataug1-1* single mutant plants to generate *ataug12* double mutants. Transformation of the T-DNA insertion single mutant *ataug3-2* with the *pHEE401E-AtAUG1 AtAUG2* construct produced an *ataug123* triple mutant. To generate *ataug1234* quadruple mutants, the *pHEE401E-AtAUG4* construct was incorporated into the Cas9-free *ataug123* triple mutants. Both Cas9-free mutant and homozygous overexpression transgenic plants were characterized based on the protocols described in [29].

DNA purified from T1 and T2 plants with CRISPR/Cas9 constructs served as the template for PCR amplification of *AtAUG1*, *AtAUG2*, and *AtAUG4* in the editing status examination. To screen Cas9-free mutants, DNA from T2 plants was extracted as the template for the Cas9 fragment amplification in order to isolate Cas9-free mutants. The primers for amplifying Cas9 fragmenst have been previously described [41].

### 4.5. Plasmid DNA Extraction, Protoplast Isolation, and Transfection

Plasmids DNA used for transcriptional activity and subcellular localization assays was isolated by using a GoldHi EndoFree Plasmid Maxi Kit (CWBIO, Taizhou, China), and protoplasts were prepared from rosette leaves of 4-week-old Col plants and transfected as previously shown [12]. For transcriptional activity assays, plasmids of the effector construct of *GD* or *GD-AtAUGs*, the activator construct of *LD-VP*, and the reporter construct of *LexA:Gal4-GUS* were co-transfected into protoplasts. Protoplasts were co-transfected with plasmids that encode the nuclear markers *NLS-RFP* and *AtAUGs-GFP* in subcellular location assays. Transfected protoplasts underwent dark treatment at RT for 20–22 h, and GFP and RFP florescence were detected using an Olympus FV1000 confocal microscope (Olympus, Tokyo, Japan), followed by GUS activity valuation using Synergy™ HT microplate reader (BioTEK, Winooski, VT, USA).

### 4.6. ABA Treatment, Root Elongation Assays, ABA Sensitivity Assays

For ABA treatment, 14-day-old seedlings of Col, the *35S:AtAUG1* transgenic plants, and the *ataug1234* quadruple mutants were immersed in 50 μM ABA for 4 h in the dark [12].

For the ABA inhibition assays, including seed germination assays, and cotyledon greening assays, 40 seeds for each genotype were sterilized and sown on 1/2 MS plates ± 1 µM ABA. After being kept in the dark at 4 °C for 48 h, the plates were transferred to a 22 °C growth chamber, where seed germination was observed on a 12-h basis, and the germination percentages were calculated. The cotyledon greening rate was calculated based on the number of green seedlings relative to the total.

In the root elongation assays for evaluating ABA inhibition, at least 100 seeds for each genotype were sterilized and geminated on vertical 1/2 MS plates. After 72 h, at least 18 seedings were selected and transferred to vertical 1/2 MS plates containing 0 µM, 5 µM, and 10 µM ABA. Root elongation and newly developed roots were observed at specific time points, and their inhibition percentage relative to the control group was calculated with the formula [(Average root length on control plates − Average root length on ABA treatment plates)/Average root length on control plates] × 100%.

### 4.7. Statistical Analysis

One-way analysis of variance (ANOVA) was used to evaluate the differences in gene expression and physiological indices between the root elongation assay and ABA cotyledon greening sensitivity assays. For multiple comparisons, different letters (a–e) indicate significant differences at *p* < 0.05. Student’s *t*-test was used to evaluate the physiological indices among ABA germination sensitivity assays. *p* < 0.05 and *p* < 0.01 represent significant and highly significant differences, respectively. In order to minimize cumulative errors, all experiments were conducted at least three independent biological and technological replicates.

## 5. Conclusions

AtAUGs are primarily localized in the nucleus and exhibit transcriptional repression activity, and their gene expression is induced by ABA. Our findings reveal that AtAUGs are involved in plant responses to ABA. *AtAUGs* overexpression significantly increased the ABA sensitivity of transgenic plants, as evidenced by reduced germination rates, lower cotyledon greening percentages, and higher root growth inhibition rates; whereas the multiple *ataugs* mutants displayed the opposite phenotype. These results indicate that AtAUGs may function redundantly in regulating plant ABA responses. Remarkably, transgenic plants overexpressing *AtAUGs* also exhibited elongated roots. Molecular mechanistic dissection revealed that the expression of PP2Cs genes that negatively regulate ABA signaling pathway, such as *PP2CA*, *HAB1*, *HAB2*, *HAI3*, *AHG1*, and *ABI2* was decreased in transgenic plants, but increased in the *ataug1234* quadruple mutant. Overall, the results of this study not only provide insights into the molecular mechanisms that AtAUGs affect ABA responses in plants, but also opens new avenues for exploring AtAUGs homologs functions in crops.

## Data Availability

All data are presented in the manuscript.

## Supplementary Materials

The following supporting information can be downloaded at: https://www.mdpi.com/article/10.3390/plants15071028/s1, Figure S1: ABA treatment effects on seed germination and seedling greening of the Col and the *35S:AtAUGs* transgenic plants. Figure S2: ABA treatment effects on seed germination and seedling greening of the Col and the *ataugs* single mutants. Figure S3: Relative expression levels of the PP2C genes, including *PP2CA*, *HAB1*, *HAB2*, *HAI3*, *AHG1* and *ABI2* in the Col, *35S:AtAUG2*, *35S:AtAUG3*, and *35S:AtAUG4* transgenic plants. Table S1: Primers used in this study.
