# Supplementary material for: AtAUGs Suppress the Expression of PP2C Genes to Redundantly Regulate ABA Responses in Arabidopsis
Source: Plants (Basel). 2026 Mar 26;15(7):1028. doi: 10.3390/plants15071028 (PMC13074224; doi:10.3390/plants15071028)
Supplement: Supplementary file 1 [file plants-15-01028-s001.zip › Supplementary figure legend.pdf]

Supplementary figure legends

Figure S1. Effects of ABA on seed germination and seedling greening of the Col wild type and the *35S:AtAUGs* transgenic plants. (**a**) Effects of ABA on seeds germination of the Col wild type and the *35S:AtAUGs* transgenic plants. Sterilized seeds of the Col wild type, and the *35S:AtAUGs* transgenic plants were plated on 1/2 MS plates with or without 1 μM ABA. Plates were kept at 4 °C in the dark for 2 days, then transferred to a growth room. Germinated seeds were recorded every 12 h, and then germination rate was calculated. Data represent mean ± SD of three replicates. *Significantly different from the Col (Student's *t*-test, *P<0.05, **P<0.01). (**b**) Image of seedlings on plates with or without ABA. Images of 20 days old seedlings were taken. (**c**) Percentage of green seedlings on plates with ABA. Green seedlings were counted and percentage of green seedlings was calculated 20 days after transferred. Data present mean ± SD of three replicates. Different letters (a-c) were used to indicate significant difference between different columns (P<0.05), as determined by one-way ANOVA.

Figure S2. Effects of ABA on seed germination and seedling greening of the Col wild type and the *ataugs* single mutants. (**a**) Effects of ABA on seeds germination of the Col wild type and the *ataugs* single mutants. Sterilized seeds of the Col wild type, and the *ataugs* single mutants were plated on 1/2 MS plates with or without 1 μM ABA. Plates were kept at 4 °C in the dark for 2 days, then transferred to a growth room. Germinated seeds were recorded every 12 h, and then germination rate was calculated. Data represent mean ± SD of three replicates. *Significantly different from the Col (Student's *t*-test, *P<0.05, **P<0.01). (**b**) Image of seedlings on plates with or without ABA. Images of 18 days old seedlings were taken. (**c**) Percentage of green seedlings on plates with ABA. Green seedlings were counted and percentage of green seedlings was calculated 18 days after transferred. Data present mean ± SD of three replicates. Different letters were used to indicate significant difference between different columns (P<0.05), as determined by one-way ANOVA.

Figure S3. Relative expression levels of the PP2C genes, including *PP2CA*, *HAB1*, *HAB2*, *HAI3*, *AHG1* and *ABI2* in the Col, *35S:AtAUG2*, *35S:AtAUG3*, and *35S:AtAUG4* transgenic plants. RNA was extracted from the plants and qRT-PCR was conducted to test the expression levels of PP2C genes. Internal reference gene was *ACT2*. Data represents SDs of three biological replicates and each experiment including three technical replicates. *Significantly different from the Col (Student's *t*-test, *$P<0.05$, **$P<0.01$).
