# Supplementary figures and images for: AtAUGs Suppress the Expression of PP2C Genes to Redundantly Regulate ABA Responses in Arabidopsis
Source: Plants (Basel). 2026 Mar 26;15(7):1028. doi: 10.3390/plants15071028 (PMC13074224; doi:10.3390/plants15071028)

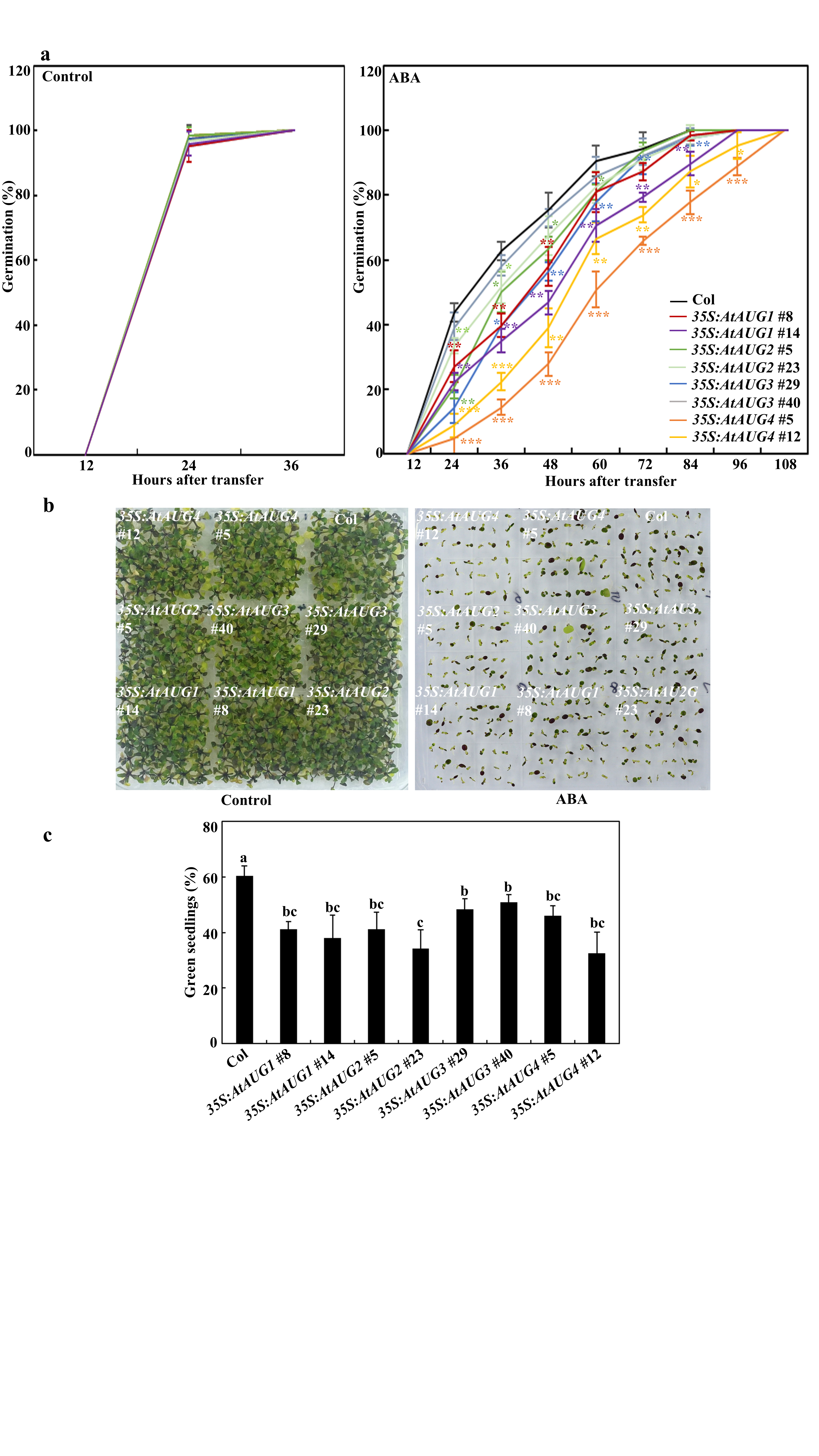

Supplement: Supplementary file 1 [file plants-15-01028-s001.zip › Figure S1.tif]

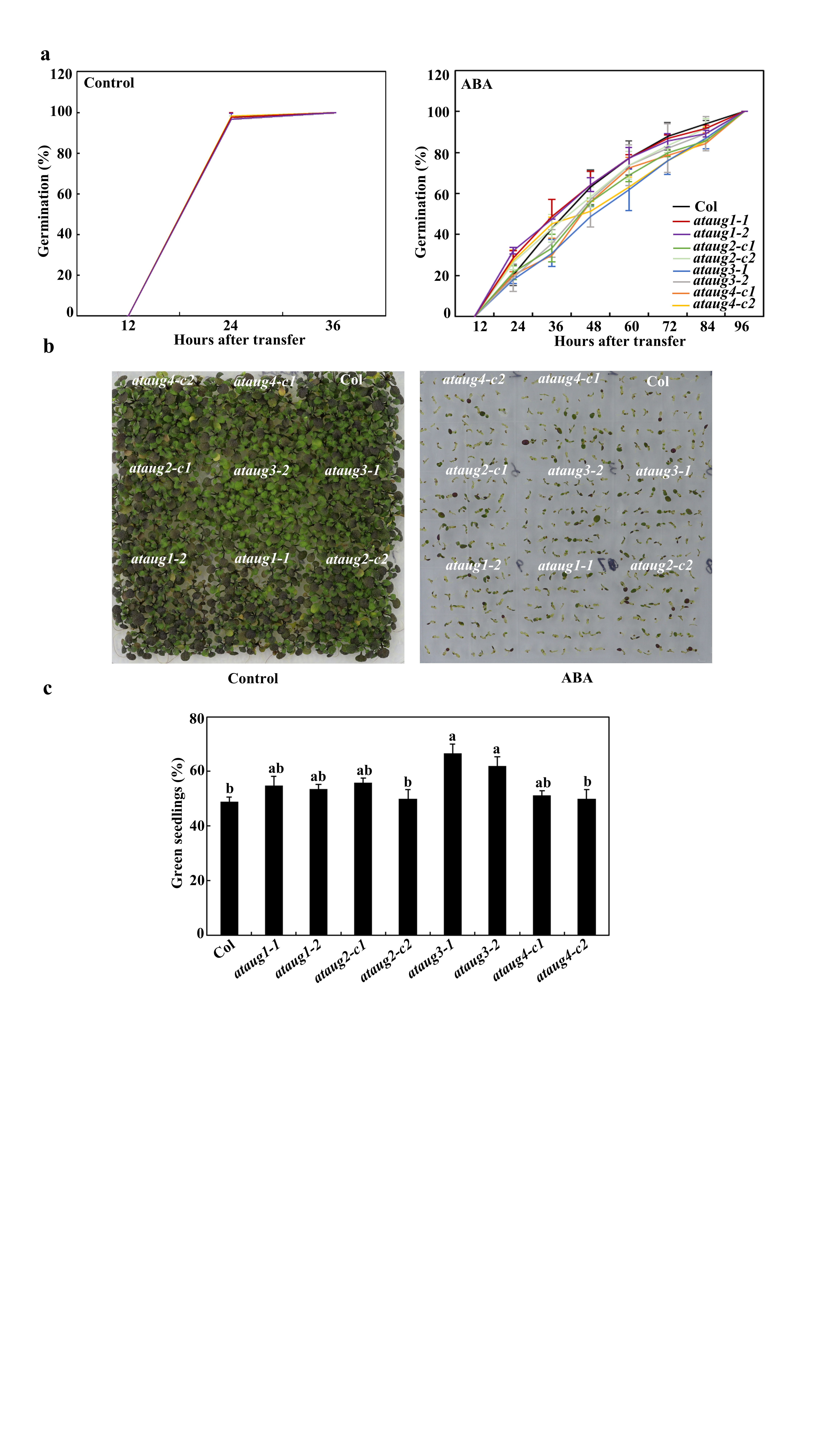

Supplement: Supplementary file 1 [file plants-15-01028-s001.zip › Figure S2.tif]

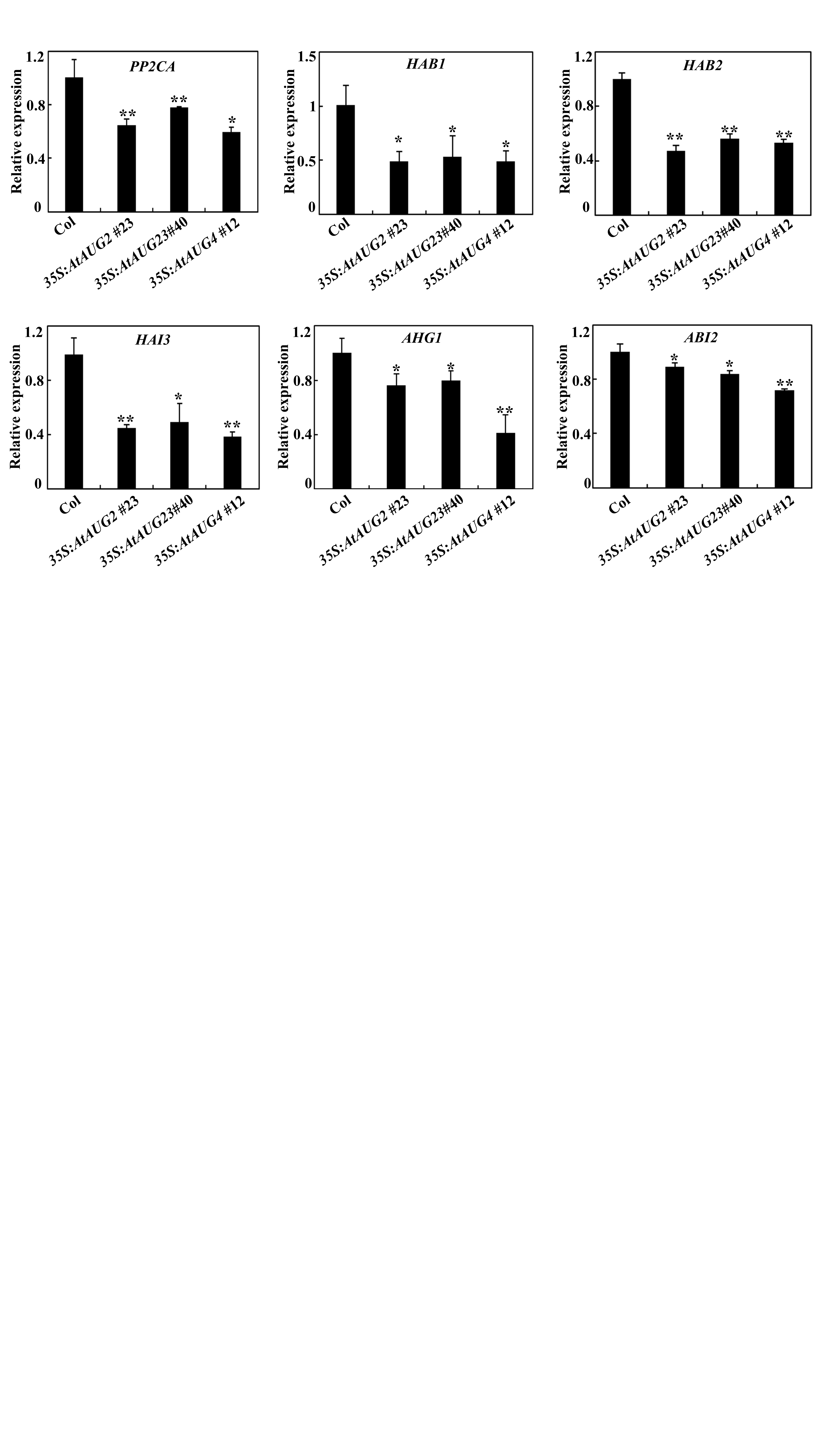

Supplement: Supplementary file 1 [file plants-15-01028-s001.zip › Figure S3.tif]
